# Maintaining Continuity of Care for Expectant Mothers in Kenya During the COVID-19 Pandemic: A Study of MomCare

**DOI:** 10.9745/GHSP-D-21-00665

**Published:** 2022-08-30

**Authors:** Teresa De Sanctis, Mary-Ann Etiebet, Wendy Janssens, Mark H. van der Graaf, Colette van Montfort, Emma Waiyaiya, Nicole Spieker

**Affiliations:** a PharmAccess Foundation, Amsterdam, The Netherlands.; b Merck & Co., Inc., Kenilworth, NJ, USA.; c Amsterdam Institute for Global Health and Development; Department of Economics; School of Business and Economics, Vrije Universiteit, Amsterdam, The Netherlands.; d PharmAccess Foundation, Nairobi, Kenya.

## Abstract

In Kenya, early coronavirus disease (COVID-19) modeling studies predicted that disruptions in antenatal care and hospital services could increase indirect maternal and neonatal deaths and stillbirths. As the Kenyan government enforced lockdowns and a curfew, many mothers-to-be were unable to safely reach hospital facilities, especially at night. Fear of contracting COVID-19, increasing costs of accessing care, stigma, and falling incomes forced many expectant mothers to give birth at home. MomCare, which primarily serves communities in remote areas and urban slums, links mothers-to-be with payers and health care providers, following a standardized pregnancy program based on World Health Organization guidelines at a predetermined cost and quality. Expectant mothers gain access to care through a mobile wallet on their feature phone (voice, text, and basic internet), and providers are paid after appropriate care is given. Within the first 3 weeks of the pandemic in Kenya, the following services were added to the MomCare bundle: emergency ambulance services during curfew hours, extended bed allowances to encourage early care, phone calls to check on mothers approaching their delivery dates and to promote the generation of a birth plan, SMS messages to inform mothers of open facilities and COVID-19 protocols, and training for clinic staff in managing COVID-19 patients and infection prevention. We compare data collected through the MomCare platform during the 6 months before the first confirmed COVID-19 case in Kenya (September 2019–February 2020) with data collected during the 6 months that followed. This study shows that care-seeking behaviors (enrollment, antenatal/postnatal care, skilled deliveries) increased for mothers-to-be enrolled in MomCare during the COVID-19 lockdowns, while quality of care and outcomes were maintained. Public health practitioners can promote interactive, patient-driven technology like MomCare to augment traditional responses, quickly linking payments with patients and providers in times of crisis.

## Abstract (precis)

During the COVID-19 pandemic in Kenya, the MomCare platform enabled care-seeking behaviors to increase and quality of care to be maintained for expectant mothers despite social, economic, and access barriers.

## Teaser

**Key Findings:**

- The outbreak of coronavirus disease (COVID-19) challenged the care-seeking behavior of expectant mothers and their access to quality health care. MomCare, a digital care bundle that links mothers-to-be with care providers and payers, quickly adapted and provided a suite of support services throughout the pandemic.
- Maintaining or improving care-seeking behaviors and quality of care for MomCare platform users shows how public health practitioners can promote interactive, patient-driven technology that can quickly link payments with patients and providers to support and empower mothers-to-be in times of crisis.

**Key Implications:**

- Program managers should consider implementing value-based health care frameworks, such as MomCare, to make care systems more outcome-driven and promote patients’ empowerment and transparency throughout the care pathway.
- Policy makers should increasingly support the adoption of mobile platforms that can promote a user-centered approach, unlock rapid scale-up over a previously organized network, ensure accountability over the care pathway, and enable rapid adaptation to unexpected changes.

## INTRODUCTION

Early coronavirus disease (COVID-19) predictions, based in part on past epidemics, painted a bleak picture for mothers and children in low- and middle-income countries.^1^ From the onset, COVID-19 affected both antenatal care (ANC) and hospital services (e.g., access to ultrasounds, skilled birth attendance, and cesarean deliveries), simultaneously jeopardizing expectant mothers’ care-seeking behavior and providers’ ability to deliver quality care.^2^^,^^3^ Analyses by the World Health Organization suggested that key health services were down by 50% or more in some African countries during the first wave of the COVID-19 pandemic (May, June, and July 2020) compared to the same 3 months in 2019.^4^ These conditions mirror those seen during the Ebola crisis in Sierra Leone, Guinea, and Liberia, where a study estimated that the decrease in use of preventive care and obstetric services (June 2014–May 2015) in Sierra Leone contributed to as many indirect deaths—stillbirth, neonatal, and maternal—as direct deaths from Ebola in the country.^5^

Social, economic, and access factors deterred some mothers from seeking care during the COVID-19 pandemic. Decreases in daily income were a consequence of the lockdown measures, especially for informal sector workers and poor households.^6^ In Nairobi, Kenya, fears over the cost of a COVID-19 quarantine or hospital stay caused a reduction in hospital admissions and outpatient visits.^7^

In the Kenyan health system, expectant mothers are free to select their preferred health care provider, be it through a private or public facility. The Kenyan National Hospital Insurance Fund offers low-cost health insurance that partially covers pregnancy-related costs. However, at a premium of around US$60 per year with penalties upon defaulted premium payments, this insurance is not affordable for many expectant mothers in remote rural areas and urban slums.^8^ A government-supported care subsidy scheme called Linda Mama is available for low-income women to cover part of their pregnancy-related costs^9^ including ANC, skilled deliveries, and postnatal care (PNC) up to the first year of life.^10^ A 2021 study of the Linda Mama program revealed that, depending on the county, between 9% and 52% of mothers pay additional out-of-pocket fees, which can be charged for medicines, tests, photocopies, supplies, or registration.^10^ Confusion regarding covered services leads some facilities to erroneously charge for covered services, such as newborn care. Delays and inconsistent reimbursement from the National Health Insurance Fund contribute to supply shortages, and some private facilities choose not to participate in Linda Mama because of low reimbursement rates. In addition, transportation costs are not covered, which limits accessibility for mothers-to-be who must travel for care.

The Kenyan National Hospital Insurance Fund offers low-cost health insurance but its premiums are out of reach for many expectant mothers in remote rural areas and urban slums.

To supplement the traditional payment schemes, MomCare is a digital platform that links expectant mothers with quality care providers through designated public and private clinics primarily located in remote rural areas or urban slums. The MomCare platform uses a digital “wallet” that either fully subsidizes an expectant mother’s care through donor funds or does so partially when an existing social insurance scheme is available (Box). Using the MomCare platform, expectant mothers have enhanced access to maternal health services and come to an agreement with their health care providers on a path of maternal care at a predetermined cost, quality, and time period. Expectant mothers then access and monitor their journey using their phones, and money is distributed to the provider once care is given.

BOX **Digital Health Wallet Eliminates Access and Economic Barriers to Health Care Services**

Most Kenyans access financial services through their mobile phone without the need for a bank account through M-PESA, a mobile money system. Building on the mobile payment infrastructure of M-PESA, CarePay (a health tech company) developed M-TIBA (https://mtiba.com/), a digital health “wallet” with dedicated funds or entitlements for health care that can be used to access care in connected clinics. MomCare operates on the M-TIBA platform.

Digital health wallets help solve both the problem of access and of payment for health care for expectant mothers who live in rural areas or urban slums. These mothers-to-be often have access to basic feature phones (voice, text, and simple internet) but lack physical addresses or health insurance, leading them to pay out of pocket for their pregnancy care, with limited insight into anticipated costs and risk of catastrophic expenditures.

The MomCare digital care bundle is a World Health Organization-based combination of maternal and child health care services and is based on the Kenyan Ministry of Health’s guidelines for obstetric and perinatal care as applicable at the time within the local setting.^11^^,^^12^ MomCare strives not only to improve outcomes (successfully mitigating risks) but also to increase the probability that risks are detected (continuously assessing risks) and mitigated early and to decrease the likelihood of complications arising.

MomCare has been developed as a digital semi-real-time mobile phone maternal and child health services solution (platform) compatible with existing information architecture. Although it is not integrated within public, private, or government information architectures, it seamlessly links with data from multiple data sources (e.g., hospital management systems, claims payment platforms, and participant surveys) to show a mother’s entire pregnancy care journey and transfer payments. Care utilization data are made available in real time across the care providers. This platform also shows payers how funds are spent. It aims to achieve transparency, accountability, and trust for all users, efficiently reaching and empowering patients and ultimately translating into better health outcomes.

The MomCare platform was developed in the Amazon Web Services cloud environment, which adheres to the highest industry standards of data protection.^13^ The platform also adheres to General Data Protection Regulation and local data protection laws. Within the MomCare architecture stored data is encrypted to further safeguard the privacy of expectant mothers. The operating costs of the platform are not covered by user fees but through external grants.

Can digital tools, such as MomCare, that take social, economic, and access factors into account, increase access to health care services during crises such as the COVID-19 pandemic? Although the benefits of digital care bundles like MomCare seem apparent in comparison to traditional approaches, studies on the effect of such digital tools on facilitating ANC and PNC during a pandemic are limited.

In this article, we examine how the MomCare bundle adapted to the sudden and unexpected challenges provided by the COVID-19 pandemic. We ask whether interventions put in place within the first 3 weeks of the lockdown in Kenya maintained access to, and the payment for, quality pregnancy care during the pandemic.

## METHODS

### Theory of Action

**FIGURE 1:**
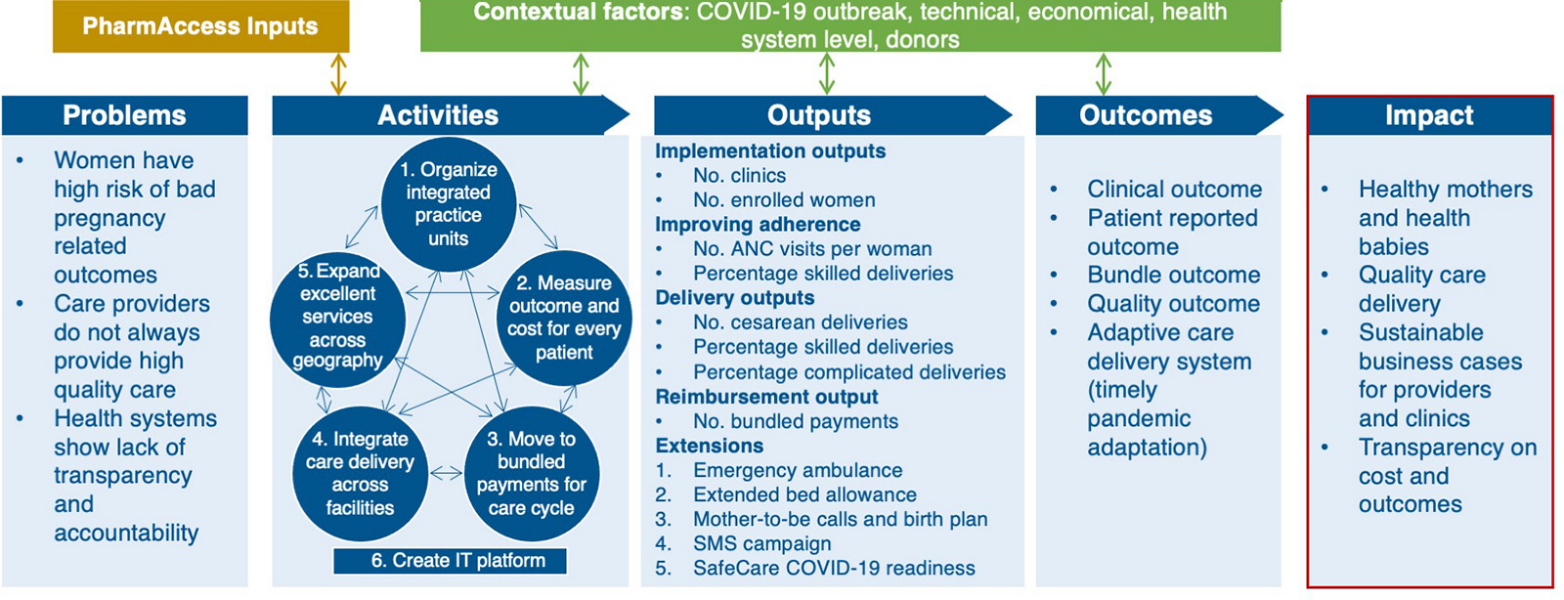
MomCare Theory of Action Abbreviations: ANC, antenatal care; COVID-19, coronavirus disease; IT, information technology; SMS, short message service.

The theory of action^14^ underlying our intervention (Figure 1) relies on MomCare’s flexible implementation of the value-based health care framework,^15^ which centers on patients’ needs. It encompasses the full cycle of care rather than single inputs, processes, and outputs—aiming to address comprehensive outcomes (both clinic and patient-reported) and to incentivize providers through appropriate reimbursement systems and smart contracts to create maximum value, defined as outcomes that matter to patients relative to the total costs of care. Examples of the value-based health care framework in MomCare^16^ include the detailed outcome measurements at the patient level, bundled payments to providers, and the adaptive digital platform in support there of (represented in the iterative Activities cycle in Figure 1). Central to the intervention is the ability to add features into the MomCare bundle, such as emergency ambulance services or extended bed allowance, that are reflected immediately and transparently across all the stakeholders. This enabled us to assess the efficacy of the newly added features; continued adherence to maternal, neonatal, and child health care services; and sustained quality of the care provided.

Central to the MomCare intervention is the ability to add features to the care bundle that are reflected immediately across all stakeholders.

### Study Setting

The MomCare bundle first rolled out in November 2017 and serves 5 counties in Kenya and Tanzania. We focused this study on the 26 MomCare clinics with the highest pre-pandemic enrollment, concentrated in 3 Kenyan counties: Nairobi (10), Kisumu (13), and Kakamega (3). The majority of MomCare clinics are privately run (54% are faith-based organizations, 38% are privately owned facilities, and the remaining 8% are public) and located in remote rural areas or urban slums. To monitor and systematically improve quality, all clinics that run the MomCare platform are backed by SafeCare, a standards-based stepwise certification methodology that rates quality of care, identifies gaps, and helps develop quality improvement plans for health care facilities in resource-constrained settings.^17^ To assess the effect that COVID-19 had on the care journey, we looked at longitudinal data from 13,443 expectant mothers enrolled in MomCare. We compared care-seeking behaviors and quality measures 6 months before the COVID-19 outbreak (September 2019–February 2020) with those seen in the study period, the first 6 months after the first case of COVID was identified in Kenya (March 2020–August 2020).

### Study Population

In Nairobi, Kisumu, and Kakamega counties, the 2015–2016 poverty headcount reported 17%, 34%, and 36% of the population, respectively.^18^ The MomCare program enrolls expectant mothers from these and other low-income populations by selecting clinics in catchment areas that mainly serve low-income women (e.g., urban slums and low-income rural areas). Baseline survey data collected from expectant mothers upon enrollment in MomCare confirm their low socioeconomic status. This sample is slightly smaller than the total number of 13,443 women included in the study period as not all survey data questions could be linked for all mothers. Of the 9,980 women linked to baseline survey data, 43% reached secondary-level education and 32% reached only primary education; 46% (of 9,840 women) cooked using wood; and 18% (of 9,946 women) reported that they or someone in their household went hungry in the past 12 months due to a lack of money for food.

### MomCare Enrollment Procedure

At the first MomCare visit (after the mother-to-be enrolls in the program), the care team reviews the consent form with the expectant mother, which includes a description of the bundle and how data are collected, limitations of liability, consent, and contact details. MomCare enrollment criteria include gestation less than 26 weeks since at this stage most of the risk of fatal outcomes can be mitigated with adequate care. However, teenagers may enroll at any time during their pregnancy, and providers have the discretion to enroll mothers-to-be identified as in high need.

**FIGURE 2:**
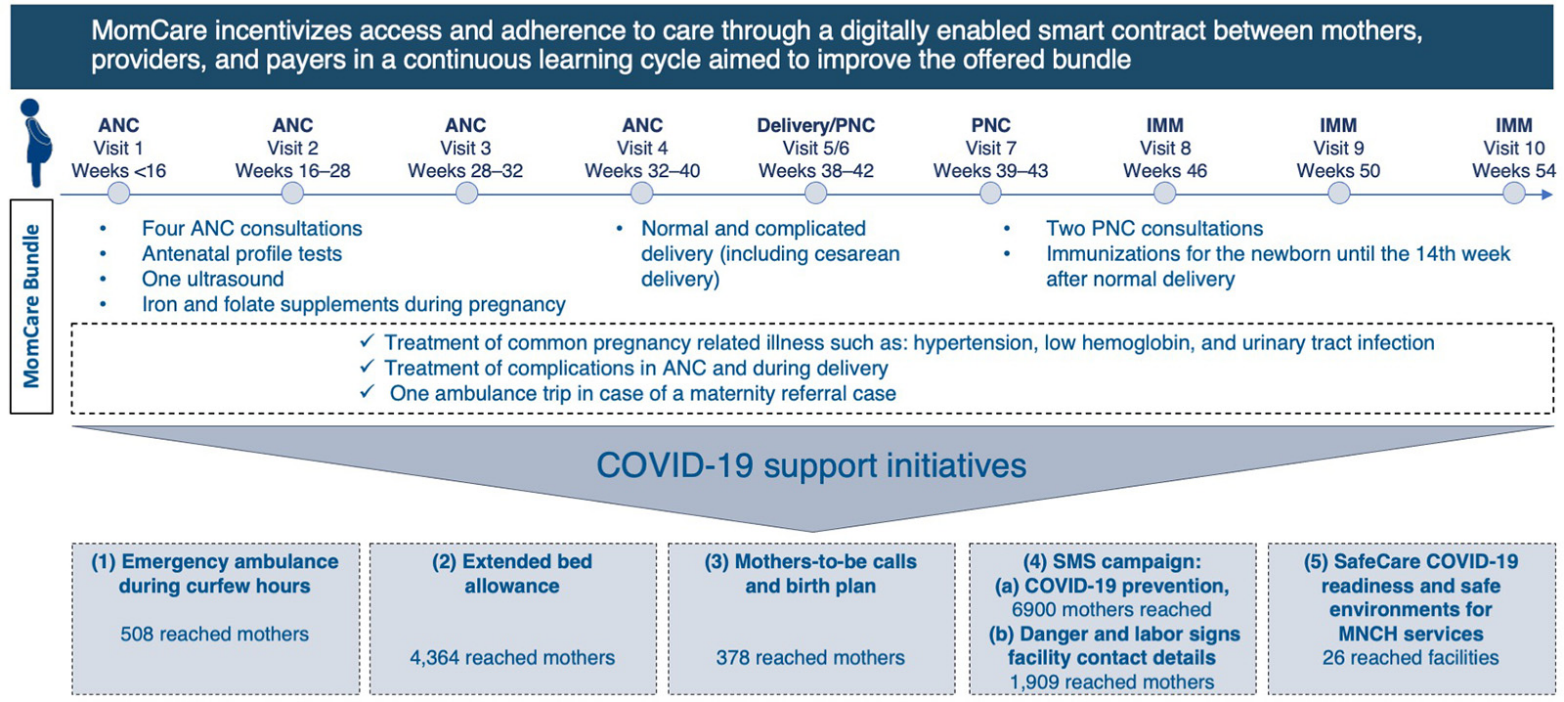
The MomCare Mother Journey, Bundle Composition, and 5 COVID-19 Support Interventions Abbreviations: ANC, antenatal care; COVID-19, coronavirus disease; IMM, immunizations; MNCH, maternal, neonatal, and child health; PNC, postnatal care; SMS, short message service.

Expectant mothers and care providers then plot their journeys—ANC, transportation, complication risk, skilled delivery, PNC, and immunizations—and digitally-enabled smart contracts are created (Figure 2, top half). These contracts are monitored by the M-TIBA health-exchange platform. They include a digital wallet that serves as a dedicated method of payment for mothers-to-be, making sure funds are available to cover the pregnancy journey. Upon arrival at a MomCare clinic, expectant mothers use their feature phones to check in. After care is received, payment is transferred instantly using mobile technology. Payers can then see transactions in real time (while the mother’s personal information is protected), fostering transparency and accountability. The patient data are also available on the MomCare app to help medical staff uncover disparities in the health journey, improve adherence to clinical guidelines, and manage high-risk patients.

### MomCare Adaptations During the COVID-19 Pandemic

On March 13, 2020, the first COVID-19-positive patient was diagnosed in Kenya,^19^ and within 3 weeks, the MomCare platform adapted to serve the changing needs of MomCare users. When governments restricted movement due to COVID-19, MomCare followed an agile cycle^20^ to adapt the platform and develop critical interventions (Figure 1). First, representatives of MomCare providers were contacted to assess their needs. Based on their responses, interventions were designed and piloted with a subset of expectant mothers and providers. Upon rapid analysis of the results, interventions were optimized before rolling them out to the full network. New features of the program included emergency ambulances during curfew hours, extended bed allowance, calls and SMS messages that reached out to mothers with fast-approaching delivery dates, and enhanced facility preparedness for COVID-19 (Figure 2, bottom half).

#### Emergency Ambulance During Curfew Hours

After the first curfew went into effect on March 27, 2020, movement was limited from dusk until dawn,^19^ causing transportation problems for expectant mothers who needed care after hours, sometimes with devastating consequences.^2^^,^^21^ While medical emergencies were meant to be exempt from curfew,^22^ the reality was different. People who violated curfew faced fines, jail, fear of altercations, and unsanctioned police brutality (especially in urban slums).^22^^–^^25^ Therefore, in addition to a daytime taxi service, MomCare provided emergency ambulance services for mothers-to-be who needed after-hours care.

#### Extended Bed Allowance

If mothers-to-be could arrive at the hospital during the day, this would eliminate the stress of traveling at night. To facilitate this MomCare extended the bed allowance from 2 to 5 days, empowering expectant mothers to seek care at the first sign that delivery was imminent.

#### Mothers-to-Be Calls and Birth Plan

For each facility, the MomCare platform generated a list of expectant mothers, between weeks 34 and 42 of gestation. The list was sorted by risk of complications and week of gestation. Midwives called mothers-to-be to check on their progress, support the generation of a birth plan, and encourage skilled delivery in a clinic staffed by a trained birth attendant. In general, facilities did not receive compensation for the airtime required to make the phone calls but rather received a payment in the form of a bonus conditional on women completing their full pregnancy journeys.

#### SMS Campaign

To assure MomCare-enrolled women (who were either pregnant or who had recently delivered) that it was safe to seek care, MomCare sent them SMS messages, informing them of government call centers and COVID-19 protocols, and directing them to dedicated MomCare interventions. Expectant mothers in their delivery period received additional information on signs of labor, danger signs, and contact details for the closest care facility.

#### SafeCare COVID-19 Readiness and Safe Environment for Maternal, Neonatal, and Child Health Services

SafeCare provided personal protective equipment and prepared health care workers at all MomCare-connected facilities. The SafeCare4Covid mobile assessment tool was used to determine the pandemic readiness of care facilities. Links were provided to digital tools and resources (e.g., guidelines, checklists, webinars, posters, patient information) in several languages, so facilities could fill gaps in knowledge.^26^ Tents dedicated to maternal, newborn, and child health services were then set up outside facilities.

Each of the newly developed support interventions aimed to mitigate the effects of the pandemic outbreak: (1) the emergency ambulance during curfew hours aimed to avoid fatal outcomes deriving from the lack of skilled support during labor; (2) the extended bed allowance aimed to avoid delivery complications arisen from delayed access to care during labor; (3) the mothers-to-be calls and birth plan aimed to increase the likelihood of medium- and high-risk mothers to attend a skilled delivery at a MomCare facility; (4) the SMS campaign intended to educate expectant mothers about the complication signs, the labor signs, and the availability of COVID-19 safe environments to access care; finally, (5) the SafeCare COVID-19 readiness and safe environment for maternal, neonatal, and child health services aimed to support the care providers in activating safe practices and environments for the expectant mothers.

### Data Collection and Analysis

First, we determined the uptake of the support interventions. Then we compared outcomes pre- and post-COVID (i.e., the control and study period, respectively) to investigate changes in care-seeking behavior and quality of care. To determine whether users of the MomCare bundle continued to seek and receive quality care during the pandemic, we examined data related to use of services, risk for complications, and outcomes (Table).

**TABLE.** Maternal and Child Health Care Outcomes in Kenya Before and During COVID-19^a^

		**Nairobi**			**Kisumu**			**Kakamega**		
		**Average**	**SD**	***P* Value**	**Average**	**SD**	***P* Value**	**Average**	**SD**	***P* Value**
Panel A. Percentage of women with a skilled/complicated/normal/cesarean/referred delivery out of all women who enrolled in MomCare during the control or study period with an expected delivery date before the end of the study period.										
Percentage of skilled deliveries	Before	12.2	3.19	0.000^b^	9.0	3.16	0.000^b^	8.2	1.72	0.000^b^
	During	17.2	4.79		18.3	8.09		16.0	4.77	
Percentage of complicated deliveries	Before	3.1	1.97	0.012^b^^,^^c^	2.0	2.10	0.067^c^	1.0	1.55	0.175^c^
	During	0.0	0.00		0.0	0.00		0.0	0.00	
Percentage of normal deliveries	Before	77.6	3.41	0.678	85.3	7.81	0.519	78.3	11.08	0.010
	During	80.0	3.58		87.3	1.03		82.0	3.90	
Percentage of cesarean deliveries	Before	19.3	3.49	0.172	12.3	5.99	0.00^b^	20.5	10.58	0.000^b^
	During	19.8	3.82		12.7	1.03		18.0	3.90	
Percentage of deliveries through referral	Before	17.8	3.13	0.329	0.8	1.17	0.001^b^	0.0	0.00	0.102^c^
	During	17.0	2.00		4.8	1.72		0.7	0.82	
Panel B. Percentage of women who were classified at some point during their pregnancy as having a medium- or high-risk pregnancy out of all women who enrolled in MomCare during the control or study period.										
Percentage of medium-risk mothers	Before	32.5	3.21	0.616	16.5	1.38	0.157	37.2	4.07	0.000^b^
	During	29.3	0.82		20.2	1.94		24.0	3.69	
Percentage of high-risk mothers	Before	35.2	2.64	0.324	43.2	3.60	0.848	36.0	4.34	0.000^b^
	During	38.5	2.59		45.2	0.41		45.5	5.09	
Panel C. Percentage of women (newborns) who received the listed diagnostic tests and supplements out of all women who enrolled in MomCare (newborns in a MomCare clinic) during the control or study period.										
Percentage of mothers with ANC profile test	Before	73.3	8.38	0.000^b^	69.0	6.90	0.368	70.3	3.78	0.000^b^
	During	60.0	16.94		70.7	2.50		52.5	11.17	
Percentage of mothers with blood pressure at each visit	Before	19.0	7.24	0.000^b^	92.7	1.75	0.991	85.7	15.63	0.000^b^
	During	42.3	7.87		91.5	1.87		83.7	10.25	
Percentage of mothers with at least one ultrasound	Before	18.2	5.49	0.000^b^	28.8	5.49	0.041^b^	25.5	7.64	0.000^b^
	During	20.7	5.32		24.3	4.89		16.7	2.88	
Percentage of mothers with folic/ferrous supplement	Before	71.3	4.84	0.133	85.8	4.92	0.671	87.3	8.55	0.279
	During	69.0	6.45		86.2	3.31		82.0	1.55	
Percentage of mothers with urine analysis at each visits	Before	60.3	9.93	0.000^b^	89.2	3.37	0.948	84.5	3.27	0.881
	During	66.0	4.77		87.0	2.53		86.7	2.34	
Percentage of mothers with oxytocin at delivery	Before	11.8	3.43	0.546	9.3	2.16	0.345	8.2	1.72	0.170
	During	12.3	1.75		11.2	1.83		10.0	1.26	
Percentage of mothers with hemoglobin test at delivery	Before	35.0	7.48	0.001^b^	51.3	3.98	0.750	44.5	3.99	0.354
	During	32.0	5.76		49.8	2.86		43.8	7.22	
Percentage of newborns with vitamin K	Before	93.7	3.44	0.610	87.0	7.01	0.692	95.2	3.60	0.033^b^
	During	86.3	3.93		87.5	1.05		82.3	3.56	

Abbreviation: ANC, antenatal care; COVID-19, coronavirus disease; SD, standard deviation; VDRL, venereal disease research laboratory.

a Results of Chi-squared test comparisons across the 6 months before the first case of COVID-19 in Kenya (September 2019–February 2020) and the following 6 months (March 2020–August 2020) (data derived as per February 15, 2021).

b Significant differences.

c *P* values based on a heteroscedastic unpaired t-test instead of a Chi-squared test since the latter is inconclusive when one of the comparison groups has mean zero throughout.

d Definitions: skilled deliveries=births that occur in a health facility connected to MomCare; complicated deliveries=births that include prepartum complications (e.g., obstructed labor) or intrapartum or postpartum hemorrhage; normal delivery=spontaneous or induced vaginal births; medium-risk mothers=mothers whose pregnancies include non-severe complications, including but not limited to asthma, urinary tract infection, candidiasis, or female genital mutilation; high-risk mothers=mothers whose pregnancies include severe complications or high-risk factors including but not limited to: pregnancies in women aged 19 years and younger, pregnancies in women aged 35 years and older; history of cesarean delivery, anemia, hypertension, diabetes, HIV, or pre-eclampsia; mothers with ANC profile test=mothers who received tests to determine blood grouping, hemoglobin (Hb), hepatitis B antigen, HIV test, VDRL test, and a urine analysis.

We used Chi-squared tests to compare data collected during the 6 months before COVID-19 (the control period, September 2019–February 2020) to the data collected during the first 6 months of the pandemic (the study period, March 2020–August 2020). All data collected during the care journey were anonymized. The analysis is performed on percentages calculated on a monthly basis to correct for the effect of birth seasonality.

#### Ethical Approval

The ethical clearance to analyze MomCare data was obtained from the Amref Health Africa Ethics and Scientific Review Committee on August 8, 2019 (approval number P679-2019).

## RESULTS

### Uptake of COVID-19 Adaptations in MomCare

**FIGURE 3:**
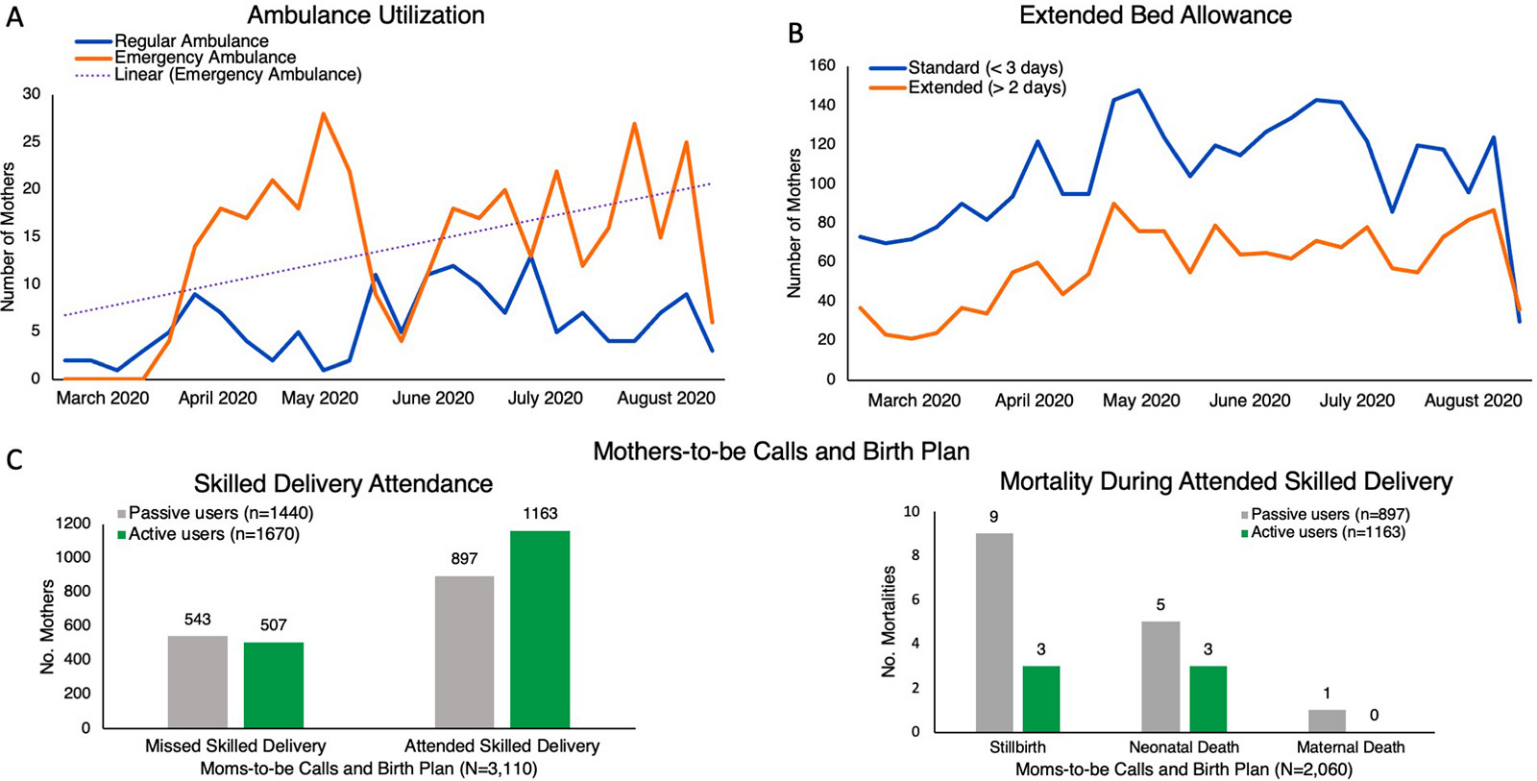
Uptake and Results of MomCare COVID-19 Response Initiatives^a^ (A) Women’s utilization of emergency inbound ambulances during curfew; (B) Women’s utilization of extended bed allowance greater than 2 days; (C) Facility-level outcomes associated with the “mothers-to-be calls and birth plan” intervention^b^ Abbreviation: COVID-19, coronavirus disease. ^a^Figures show results over the study period March 2020–August 2020. ^b^Active users are health care providers who logged more than 20 page views of mothers-to-be data; passive users are health care providers who logged 20 or less page views of mothers-to-be data.

Figure 3 shows the uptake and utilization results of the newly introduced COVID-19 interventions in MomCare. In total, 508 expectant mothers took advantage of the emergency ambulance services during curfew hours (Figure 3a). The extended bed allowance helped 4,364 mothers-to-be (Figure 3b). Compared to usage seen during the 6 months before the COVID-19 outbreak (average of 13.8 expectant mothers per week), more than 4 times as many users of the MomCare bundle extended their hospital stay more than 2 days (average of 57.8 expectant mothers per week).

Midwives conducted 378 calls with mothers-to-be to discuss their upcoming delivery. While data from the “mothers-to-be calls and birth plan” intervention are limited and not statistically powered, they suggest that when health care providers actively engage with the MomCare platform, it may have a positive effect on outcomes. Here, active users are defined as health care providers who logged more than 20 page views of mothers-to-be data within the study period, while passive users logged 20 views or less. Active users experienced an increase in skilled deliveries at their facility, and fewer adverse events compared to passive users (Figure 3c, left- and right-panel, respectively). Overall, the group of active users contains more urban care providers (64%) compared to the passive users (while urban care providers correspond to 13% of the sample).

Finally, through the SMS campaign, the platform sent 6,900 SMS messages related to COVID-19 prevention, including an SMS blast to all mothers who had ever used MomCare. Moreover, MomCare sent 1,909 messages specifically to expectant mothers in their delivery period with delivery-tailored information.

### Care-Seeking Behavior

**FIGURE 4:**
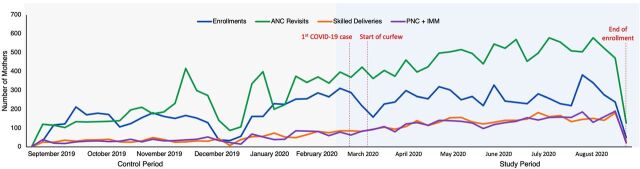
MomCare Use Pre- and Post-COVID-19,^a^ September 2019–August 2020 Abbreviations: ANC, antenatal care; COVID-19, coronavirus disease; IMM, immunizations; PNC, postnatal care. ^a^MomCare use across the 6 months before the first case of COVID-19 in Kenya (September 2019–February 2020) and the following 6 months (March 2020–August 2020). Data derived February 15, 2021.

During the COVID-19 pandemic, the MomCare program continued to expand. As a result, the 3 counties saw an increased number of enrollments, ANC revisits, skilled deliveries, and PNC visits (including immunizations) (Figure 4).

During the pandemic the 3 study counties saw increased enrollments, ANC revisits, skilled deliveries, and PNC visits.

Compared to the pre-COVID period, the percentage of skilled deliveries increased significantly as well in all 3 counties, while the percentage of MomCare users with complicated deliveries dropped—significantly so in Nairobi and Kisumu counties (Table, Panel A). This suggests that MomCare was effective in ensuring maintained access to skilled delivery services, efficiently communicating with mothers-to-be throughout their pregnancies, while aiding providers in organizing safe environments for delivery. In addition, the percentage of mothers who delivered through referral increased significantly in Kisumu, illustrating the strength of connections between facilities in the program.

### Risky Pregnancies

In Kakamega county, the most rural of the 3, the percentage of medium-risk mothers-to-be significantly decreased, while the percentage of high-risk mothers increased (Table, Panel B). For some mothers, the MomCare interventions may not have been enough to counteract the weight of the pandemic. On the other hand, we also observe a shift from cesarean to normal deliveries in Kakamega (Table, Panel A), suggesting that the COVID-19 support interventions may have enhanced communication between at-risk mothers and providers, leading to timely actions, and making it easier for providers to promptly handle at-risk pregnancies, likely preventing some high-risk pregnancies from escalating.

### Quality of Care

Panel C of the Table shows that the percentage of pregnant women with ANC profile tests decreased significantly in Nairobi and Kakamega counties. The economic challenges faced by the informal sector during the curfew could partly explain this drop.^6^ Simultaneous increases in transportation costs and decreases in availability of care due to government-regulated transit restrictions^27^ were obstacles that likely prevented some expectant mothers from seeking timely ANC. The stigma of a COVID-19 diagnosis also kept some from seeking facility-based care. COVID-19 testing and hospital admissions for common ailments declined, as people feared a positive diagnosis would brand them as outcasts.^28^ Reports also told of women who delayed care out of fear of contracting COVID-19 at the hospital.^2^^,^^21^^,^^29^

In Nairobi county, the percentage of mothers-to-be who received blood pressure monitoring increased substantially, as did the percentage of women with at least 1 ultrasound and urine analysis at each visit (Table, Panel C). The additional support that MomCare provided to these clinics during the pandemic may have ensured that providers had safe workspaces and adequate staff. Still, hemoglobin tests at delivery declined, suggesting that challenges were not fully overcome. The quality of care in Kisumu was largely maintained during the pandemic, diagnostic tests were performed, and supplements were provided, as before. An exception we observe is a decline in the percentage with at least 1 ultrasound.

In contrast, Kakamega county seems to have been the most affected by COVID-19. It also showed a significant decrease in the percentage of newborns receiving vitamin K supplementation to prevent brain hemorrhage shortly after birth. Widespread challenges to the supply chain have been reported across Kenya since COVID-19 shutdowns began,^30^^,^^31^ but a survey conducted in July 2019 showed that fluctuations in the availability of medicines for mothers and newborns may have been common before the pandemic.^32^ The presented results might represent the supporting evidence of the aggravated challenges that the pandemic unleashed on the supply chain, generating a shortage of drugs, especially in more remote, rural regions such as Kakamega.

In all 3 counties, the percentage of mothers-to-be who were provided with folate and iron supplements and oxytocin at delivery remained relatively constant (Table, Panel C).

## DISCUSSION

The sustained enrollment and increased skilled deliveries that were observed during the pandemic were critical not only for mothers and babies but also for clinics struggling to stay in business. Over time, the pandemic might add greater stress to the health care delivery system as well as to expectant mothers’ health-seeking behavior, as sustained economic hardships heighten anxiety and force hard decisions for Kenyan families. MomCare provides a level of transparency that lowers stress for mothers and providers. By eliminating the fear that care cannot be paid for and increasing awareness of warning signs, MomCare empowers mothers-to-be to seek timely care. Previous research, such as the 3-delay model, has shown that decreasing delays in seeking emergency care can lower maternal and infant mortality.^33^ Thanks to the dedicated support interventions during the pandemic outbreak, pregnant mothers within the MomCare network were able to continue accessing care while care providers struggled to maintain access to care for other patient populations such as those with noncommunicable diseases, leading in most cases, to increased risk of bad outcomes.

MomCare eliminates fear that care cannot be paid for and increases awareness of pregnancy warning signs.

Early models in Kenya projected that disruption of care during the COVID-19 pandemic could lead to as many as 2,120 additional child deaths and 196 additional maternal deaths during each month of the pandemic,^1^ and there have been lawsuits and tragic deaths related to the implementation of COVID-19 restrictions.^32^^–^^34^ In the MomCare experience, digital interventions communicated vital health information and promoted care-seeking behaviors during a pandemic, helping to maintain consistency in care and encourage progress toward lowering maternal and infant deaths in the region.

### Challenges

The main challenges faced during the development of the COVID-19 support interventions were the design and validation of usable and viable solutions with the mothers and care providers, operational feasibility of the designed solutions across the facilities network, and timely rollout of the additional features. These challenges were overcome through an agile^20^ development approach utilizing frequent interaction cycles with the end users that enabled deployment of the support interventions in a relatively short time (3 weeks).

We overcame development challenges through an agile approach utilizing frequent interaction cycles with the end users.

### Limitations

One limitation of this study of MomCare was the restricted data related to health outcomes—maternal deaths, neonatal deaths, and stillbirths. While data from the mothers-to-be call and birth plan intervention suggest that MomCare improved outcomes, future studies that extend the time period and dataset could be helpful. Additional studies could also compare outcomes at MomCare clinics to those seen at public or private facilities that don’t run the MomCare platform (since our data do not allow insights on mothers delivering outside MomCare)—with the note of caution that an adequate comparison group for MomCare should consist of equally low-income and disadvantaged mothers-to-be with low access to maternal health care throughout their journey.

Another limitation concerns the incremental nature and relatively short time frame of the dataset. A standardized, longitudinal sample collected over a longer period would enable an interrupted time series comparison, useful to complement the implementation learnings with an evaluation of the impact on utilization and outcomes. We do not have the right data available for such a causal analysis. First, a longer time frame would not cover all current clinics, due to the gradual scaling of MomCare; only a few clinics were included in the intervention before our study period starting 6 months before the pandemic. Second, the number of women who were enrolled in MomCare (as captured in the utilization patterns) gradually increased as well because the intervention itself expanded within the participating clinics. Therefore, we presented a descriptive analysis, focusing on how ad hoc, well-defined additions of supporting interventions may minimize the effect of the pandemic on adherence to care and the quality of services. For a more detailed investigation of pre-COVID utilization patterns in a comparable sample, we refer to Aksünger et al. (2022).^35^

## CONCLUSION AND NEXT STEPS

Partnering with care providers, MomCare identified barriers posed by COVID-19 and worked through them, empowering new enrollment and encouraging users of the care bundle to complete ANC visit schedules, seek skilled deliveries, and return for PNC/immunization appointments. The combination of interventions—access to information, transportation, quality care, and affordable care—empowered expectant mothers to pursue the care they were entitled to while providing a platform for them to make birthing plans and foster trusting relationships with care providers.

The MomCare platform demonstrated how health systems and public health practitioners can use mobile connections and emerging technologies—algorithms, computing power, and feedback tools like mobile apps—to give vulnerable individuals the tools they need to take charge of their health. It showed that mobile platforms can be rapidly scaled over a previously organized network, focusing enhancements on the expressed needs of users, while adding transparency and accountability throughout the care pathway. Expectant mothers were engaged through technology, and providers maintained vital services while receiving prompt, traceable payment.

There is a wide opportunity for public health practitioners to promote interactive, patient-driven technology that augments traditional institutional responses. Such collaborations make health systems more human-centered, more adaptive, and therefore better able to quickly adapt to unforeseen crises like future pandemics. Using data and interventions, mobile connections, and emerging technology can create opportunities to personalize care, enhance transparency, and protect vulnerable individuals.

As MomCare enters the next stages of development, the focus will be on enhancing scalability and sustainability. The first step will be scaling up the program and increasing its integration into public and private health systems in Kenya and beyond, by making it easier to connect the MomCare platform to the growing number of health information and payment systems that gather the required data. Second, to enhance the sustainability of the program, increased focus will be placed on leveraging existing social and private insurance funds and universal health coverage initiatives available in the market, as well as the potential for expectant mothers to contribute depending on their socioeconomic status. Third, future MomCare enhancements will continue the drive toward value-based care, supporting care reimbursements that are increasingly dependent on quality outcomes rather than fee-for-service or capitation fees. In doing so, digital interventions like the MomCare initiative can support care systems to become more empowering for patients, more transparent for payers, and more effective and outcome-driven for providers.

## Notes

**Peer Reviewed**

**First published online:** August 24, 2022.

## References

[1] Roberton T Carter ED Chou VB. Early estimates of the indirect effects of the COVID-19 pandemic on maternal and child mortality in low-income and middle-income countries: a modelling study. Lancet Glob Health. 2020;8(7):e901–e908. 10.1016/S2214-109X(20)30229-1. 32405459 PMC7217645

[2] Kimani RW Maina R Shumba C Shaibu S. Maternal and newborn care during the COVID-19 pandemic in Kenya: re-contextualising the community midwifery model. Hum Resour Health. 2020;18(1):75. 10.1186/s12960-020-00518-3. 33028347 PMC7539267

[3] Ahmed SAKS Ajisola M Azeem K; Improving Health in Slums Collaborative. Impact of the societal response to COVID-19 on access to healthcare for non-COVID-19 health issues in slum communities of Bangladesh, Kenya, Nigeria and Pakistan: results of pre-COVID and COVID-19 lockdown stakeholder engagements. BMJ Glob Health. 2020;5(8):e003042. 10.1136/bmjgh-2020-003042. 32819917 PMC7443197

[4] COVID-19 hits life-saving health services in Africa. World Health Organization Regional Office for Africa. November 5, 2020. Accessed March 18, 2021. https://www.afro.who.int/news/covid-19-hits-life-saving-health-services-africa

[5] Sochas L Channon AA Nam S. Counting indirect crisis-related deaths in the context of a low-resilience health system: the case of maternal and neonatal health during the Ebola epidemic in Sierra Leone. Health Policy Plan. 2017;32(Suppl 3):iii32–iii39. 10.1093/heapol/czx108. 29149310

[6] Janssens W Pradhan M de Groot R Sidze E Donfouet HPP Abajobir A. The short-term economic effects of COVID-19 on low-income households in rural Kenya: an analysis using weekly financial household data. World Dev. 2021;138:105280. 10.1016/j.worlddev.2020.105280

[7] Njue D. Declining health service use in Nairobi has health implications beyond COVID-19, 2020. *Africa at LSE* blog. June 25, 2020. Accessed February 5, 2021. https://blogs.lse.ac.uk/africaatlse/2020/06/25/declining-health-service-use-in-nairobi-has-health-implications-beyond-covid-19/

[8] Individuals. National Hospital Insurance Fund. Accessed October 13, 2021. https://blog.nhif.or.ke/website/individuals/

[9] Linda Mama Hospitals. National Hospital Insurance Fund. Accessed October 13, 2021. https://blog.nhif.or.ke/website/linda-mama-hospitals/

[10] Orangi S Kairu A Ondera J. Examining the implementation of the Linda Mama free maternity program in Kenya. Int J Health Plann Manage. 2021;36(6):2277–2296. 10.1002/hpm.3298. 34382238 PMC9290784

[11] World Health Organization (WHO). *WHO Recommendations on Antenatal Care for a Positive Pregnancy Experience*. WHO; 2016. Accessed May 18, 2021. https://www.who.int/publications/i/item/9789241549912/28079998

[12] Republic of Kenya. Ministry of Health (MOH). Department of Family, Division of Reproductive and Maternal Health. *National Guidelines on Quality Obstetric and Perinatal Care*. MOH; 2020.

[13] Security and compliance. Amazon Web Services. Accessed December 3, 2021. https://docs.aws.amazon.com/whitepapers/latest/aws-overview/security-and-compliance.html

[14] Goeschel CA Weiss WM Pronovost PJ. Using a logic model to design and evaluate quality and patient safety improvement programs. Int J Qual Health Care. 2012;24(4):330–337. 10.1093/intqhc/mzs029. 22745358

[15] Porter ME. What is value in health care? N Engl J Med. 2010;363(26):2477–2481. 10.1056/NEJMp1011024. 21142528

[16] Leapfrog to Value. *Leapfrog to Value: How Nations Can Adopt Value-Based Care on the Path to Universal Health Coverage*. Leapfrog to Value; 2019. https://www.leapfrogtovalue.org/flagship-report

[17] The future of quality in healthcare. SafeCare. Accessed November 19, 2020. https://www.safe-care.org/

[18] Kenya Institute for Public Policy Research and Analysis (KIPPRA). *Kenya Economic Report 2020: Creating an Enabling Environment for Inclusive Growth in Kenya*. KIPPRA; 2020. Accessed October 13, 2021. https://kippra.or.ke/wp-content/uploads/2021/02/Kenya-Economic-Report-2020.pdf

[19] Kenya COVID-19 Economic Tracker. Accessed February 10, 2021. https://www.kenyacovidtracker.org/

[20] Beck K Beedle M van Bennekum A. Manifesto for agile software development. Accessed May 18, 2021. http://agilemanifesto.org

[21] Vellekoop M Acholla M Hanne J. Falling through the cracks: COVID-19 and the rise of maternal deaths in Africa. Think Global Health. September 21, 2020. Accessed November 21, 2020. https://www.thinkglobalhealth.org/article/falling-through-cracks-covid-19-and-rise-maternal-deaths-africa

[22] Okeyo V Kabale N Maundu P Oudia R. The COVID-19 nightmare for pregnant women. *Daily Nation*. April 14, 2020. Accessed November 28, 2020. https://nation.africa/kenya/healthy-nation/the-covid-19-nightmare-for-pregnant-women-647422

[23] Odula T. Pregnant women at risk of death in Kenya’s COVID-19 curfew. *Associated Press*. July 25, 2020. Accessed December 1, 2020. https://apnews.com/article/virus-outbreak-ap-top-news-health-africa-international-news-2e1a7d8b8401e4c06df52085994cf4ba

[24] Kimani K. COVID-19 – “New” criminal offences in Kenya. JD Supra. April 17, 2020. Accessed May 18, 2021. https://www.jdsupra.com/legalnews/covid-19-new-criminal-offences-in-kenya-76521/

[25] Ateva E. COVID-19 curfew restrictions impact reproductive, maternal, and newborn health and rights worldwide. White Ribbon Alliance. June 8, 2020. Accessed July 28, 2022. https://web.archive.org/web/20220315205405/https://www.whiteribbonalliance.org/2020/06/08/covid-19-curfew-restrictions-impact-women-and-newborns-worldwide/

[26] Downloads and resources. SafeCare. Accessed February 10, 2021. https://www.safe-care.org/resources

[27] Bird J Kriticos S Tsivanidis N. Impact of COVID-19 on public transport. International Growth Centre. August 6, 2020. Accessed November 28, 2020. https://www.theigc.org/blog/impact-of-covid-19-on-public-transport/

[28] Waruru M. Stigma blots Kenya’s COVID-19 war – some patients fearful of seeking treatment. Health Policy Watch. September 8, 2020. Accessed February 10, 2021. https://healthpolicy-watch.news/stigma-blots-kenyas-covid-19-war-as-country-abandons-contract-tracing/

[29] Oluoch-Aridi J Chelagat T Nyikuri MM. COVID-19 effect on access to maternal health services in Kenya. Front Glob Womens Health. 2020;1:599267. 10.3389/fgwh.2020.599267. 34816169 PMC8593959

[30] Banga K Keane J Mendez-Parra M Pettinotti L Sommer L. Africa Trade and COVID-19: The Supply Chain Dimension. Working paper 586. ODI; 2020. Accessed November 28, 2020. https://www.odi.org/publications/17248-africa-trade-and-covid-19-supply-chain-dimension

[31] Covid-19 reversing maternal gains. Nation.Africa. April 13, 2020. Accessed December 4, 2020. https://nation.africa/kenya/gender/covid-19-reversing-maternal-gains-647266

[32] Ooms GI Kibira D Reed T van den Ham HA Mantel-Teeuwisse AK Buckland-Merrett G. Access to sexual and reproductive health commodities in East and Southern Africa: a cross-country comparison of availability, affordability and stock-outs in Kenya, Tanzania, Uganda and Zambia. BMC Public Health. 2020;20(1):1053. 10.1186/s12889-020-09155-w. 32620159 PMC7333276

[33] Calvello EJ Skog AP Tenner AG Wallis LA. Applying the lessons of maternal mortality reduction to global emergency health. Bull World Health Organ. 2015;93(6):417–423. 10.2471/BLT.14.146571 26240463 PMC4450708

[34] Namubiru L Wepukhulu KS. African governments warned of lawsuits for maternal deaths under COVID-19 lockdowns. Open Democracy. July 16, 2020. Accessed November 28, 2020. https://www.opendemocracy.net/en/5050/african-governments-warned-lawsuits-maternal-deaths-covid-lockdowns/

[35] Aksünger N De Sanctis T Waiyaiya E van Doeveren R van der Graaf M Janssens W. What prevents pregnant women from adhering to the continuum of maternal care? Evidence on interrelated mechanisms from a cohort study in Kenya. BMJ Open. 2022;12(1):e050670. 10.1136/bmjopen-2021-050670. 35039285 PMC8765038

